# Supplementary material for: Discovery of a New Drug-like Series of OGT Inhibitors by Virtual Screening
Source: Molecules. 2022 Mar 19;27(6):1996. doi: 10.3390/molecules27061996 (PMC8950328; doi:10.3390/molecules27061996)

Discovery of a New Drug-Like Series of OGT Inhibitors by Virtual Screening.

Elena M. Loi[1,2], Tihomir Tomašič[1], Cyril Balsollier[2,1], Kevin van Eekelen[2], Matjaž Weiss[1], Martina Gobec[1], Matthew G. Alteen[3], David J. Vocadlo[3], Roland J. Pieters[2], Marko Anderluh[1]

[1]*Department of Pharmaceutical Chemistry, Faculty of Pharmacy, University of Ljubljana, Ljubljana, 1000, Slovenia*

[2]*Department of Chemical Biology & Drug Discovery, Utrecht Institute for Pharmaceutical Sciences, Utrecht University, 3584 CG Utrecht, The Netherlands*

[3]*Department of Chemistry, Simon Fraser University, Burnaby, BC, V5A 1S6, Canada*

Supplementary information

Table S1. List of most potent OGT inhibitors reported to date and their properties.

Name	Structure	Inhibitory parameter	Issue	Reference
**Substrate analogues**				
**Ac_4-5S-GlcNAc**	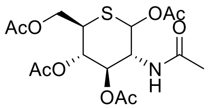	EC_{50} = 5 μM	Cell permeable<br>Off-target effects<br>Poor aqueous solubility	Dorfmueller, H. C. et al. Amino Acids 40, 781–792 (**2011**).
**5S-GlcNHex**	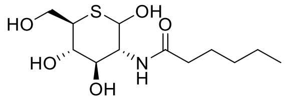	Similar to Ac_4-5S-GlcNAc	Cell permeable<br>Off-target effects	Liu, T. W. et al. Angew. Chemie - Int. Ed. 57, 7644–7648 (**2018**).
**Ac_4-ES1**	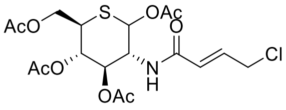	Similar to Ac_4-5S-GlcNAc	Cell permeable<br>Covalent inhibitor<br>Selective	Worth, M. et al. Chem. Commun. 55, 13291–13294 (**2019**).
**High-throughput and virtual screening identified inhibitors**				
**OSMI-1**	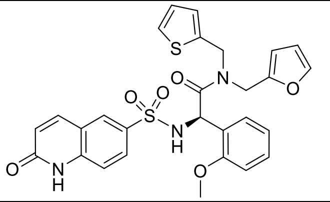	IC_{50} = 2.7 μM	Cell permeable<br>Off-target effects	Ortiz-Meoz, R. F. et al. ACS Chem. Biol. 10, 1392–1397 (**2015**).

**OSMI-2**<br>**OSMI-3**<br>**OSMI-4**	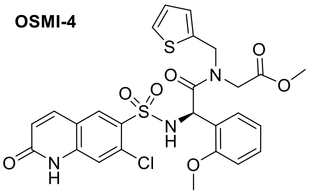	OSMI-2 (acid form) K_D = 140 nM<br>OSMI-3 (acid form) K_D = 5 nM<br>OSMI-4 (acid form) K_D = 8 nM	Very potent<br>Cell permeable<br>Specific	Martin, S. E. S. et al. J. Am. Chem. Soc. 140, 13542–13545 (**2018**).
**L01**	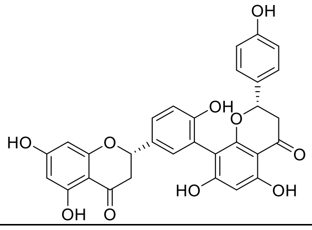	IC_{50} = 21.8 µM	Cell permeable<br>Natural product<br>Off-target effects	Liu, Y. et al. Sci. Rep. 7, 12334 (**2017**)
**APNT and APBT**	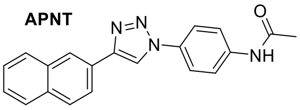	APNT:<br>IC_{50} = 66.7 µM<br>APBT<br>IC_{50} = 139 µM	Cell permeable<br>Non-competive<br>Poor aqueous solubility<br>Low potency	Wang, Y.- et al. J. Med. Chem. 60, 263–272 (**2017**).
**LQMed 330**	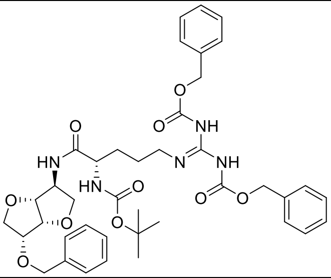	IC_{50} = 11.7 µM	Not selective<br>Unknown solubility and permeability	Albuquerque, S. O. et al. Eur. J. Pharm. Sci. 154, 105510 (**2020**)
**OGT inhibitors from our group**				
**F20**	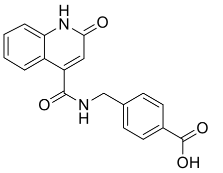	IC_{50} = 116.0 µM	Cell permeable<br>Low potency	Zhang, H. et al. MedChemComm 9, 883–887 (**2018**).
**6b**	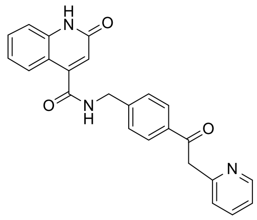	IC_{50} = 144.5 µM	Low potency	Weiss, M. et al. Front. Chem. 9, 205 (**2021**).

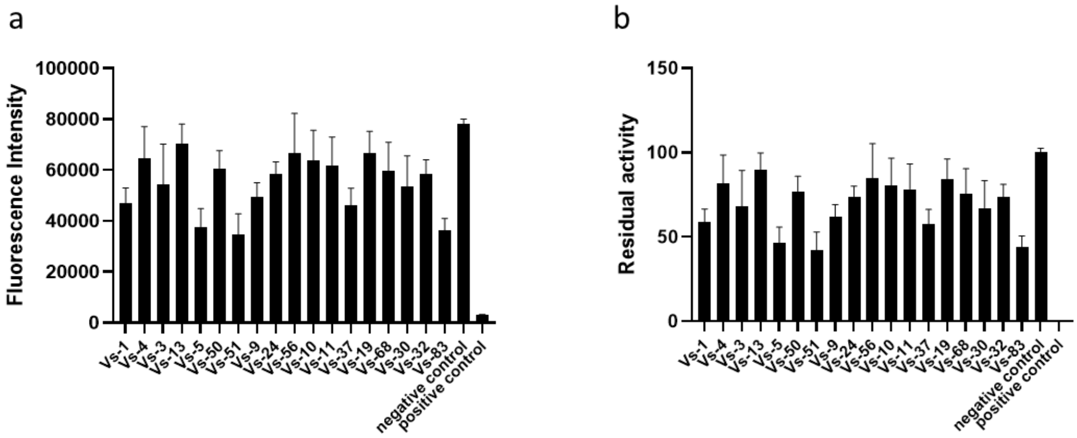

Figure S1. Results of the screening with OGT fluorescent activity assay. The compounds were screened at a fixed concentration of 100 µM in two independent experiments. The results are expressed as fluorescence intensity values (a) or normalized residual activity values (b), with respect to the negative (DMSO) and positive (OSMI-4) controls.

Table S2. Details of the commercially available compounds screened in this study.

Name	Structure	Vendor	ID	Purity declared by vendor
**Vs-1**	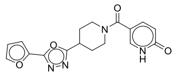	Life Chemicals	F5596-0429	≥90%
**Vs-4**	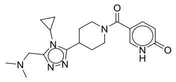	ChemBridge	87364924	≥85%
**Vs-3**	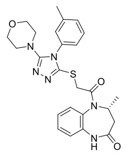	Enamine	Z92480524	≥90%
**Vs-13**	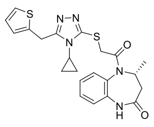	Enamine	Z90105807	≥90%
**Vs-24**	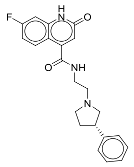	ChemBridge	39842207	≥85%
**Vs-30**	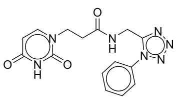	ChemBridge	72669148	≥85%

**Vs-5**	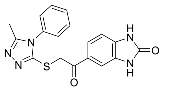	Enamine	Z24159125	≥90%
**Vs-50**	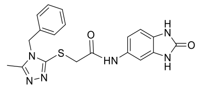	Enamine	Z24541217	≥90%
**Vs-51**	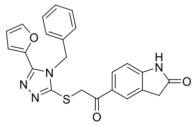	Enamine	Z65683626	≥90%
**Vs-9**	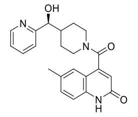	ChemBridge	87867766	≥85%
**Vs-56**	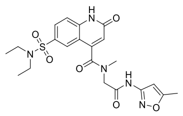	Enamine	Z220335192	≥90%
**Vs-32**	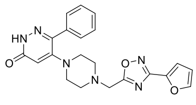	ChemBridge	82571109	≥85%
**Vs-10**	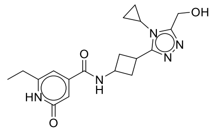	ChemBridge	41869522	≥85%
**Vs-11**	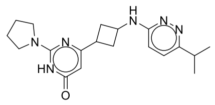	ChemBridge	46452758	≥85%
**Vs-37**	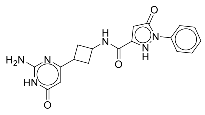	ChemBridge	53010325	≥85%
**Vs-19**	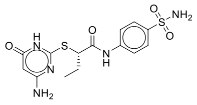	ChemBridge	7555184	≥90%
**Vs-68**	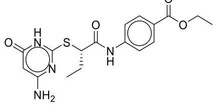	ChemBridge	7915441	≥90%
**Vs-83**	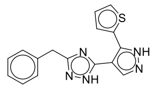	ChemBridge	40085129	≥85%

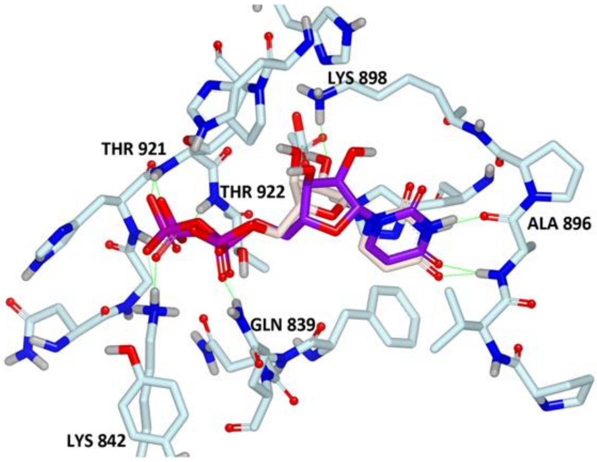

Figure S2. Validation of the docking experiment: overlay of the co-crystallized (beige) and re-docked (purple) ligand UDP. PDB entry: 4N39. RMSD value: 1.45.

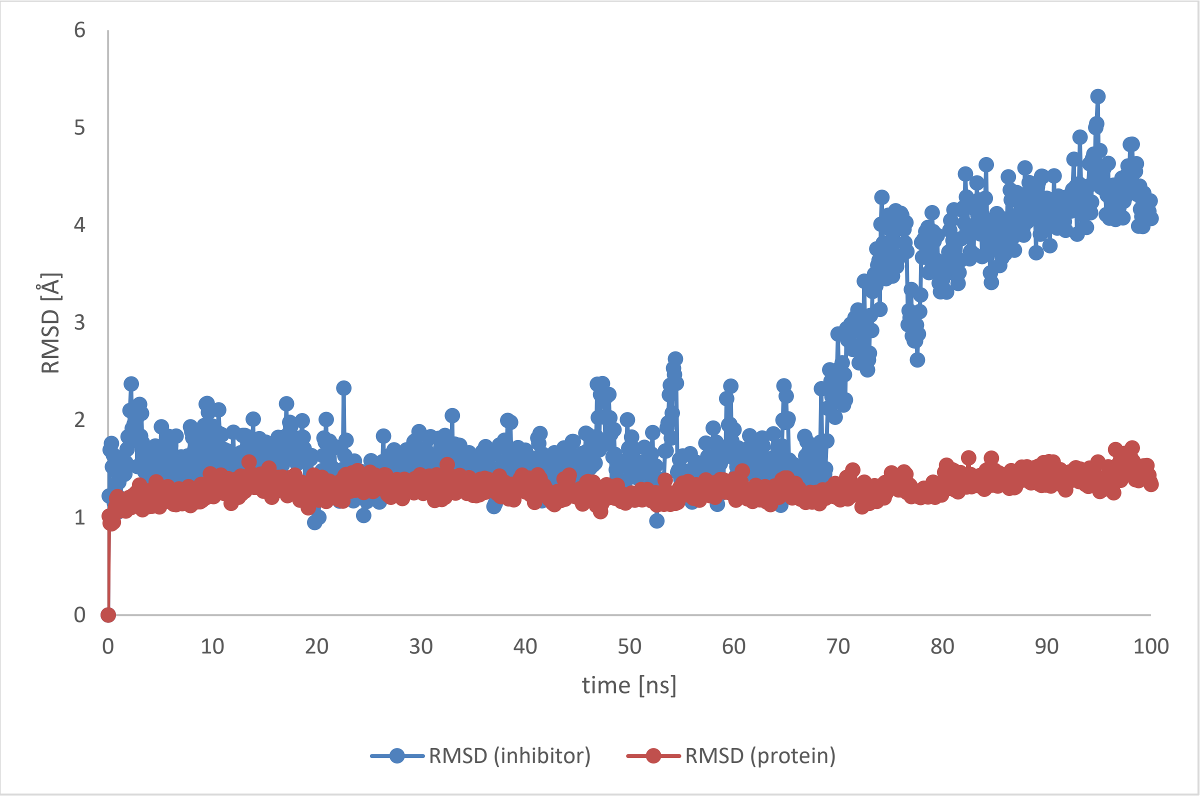

Figure S3. Protein (in red) and ligand (in blue) RMSD values during the 100 ns molecular dynamics simulation.

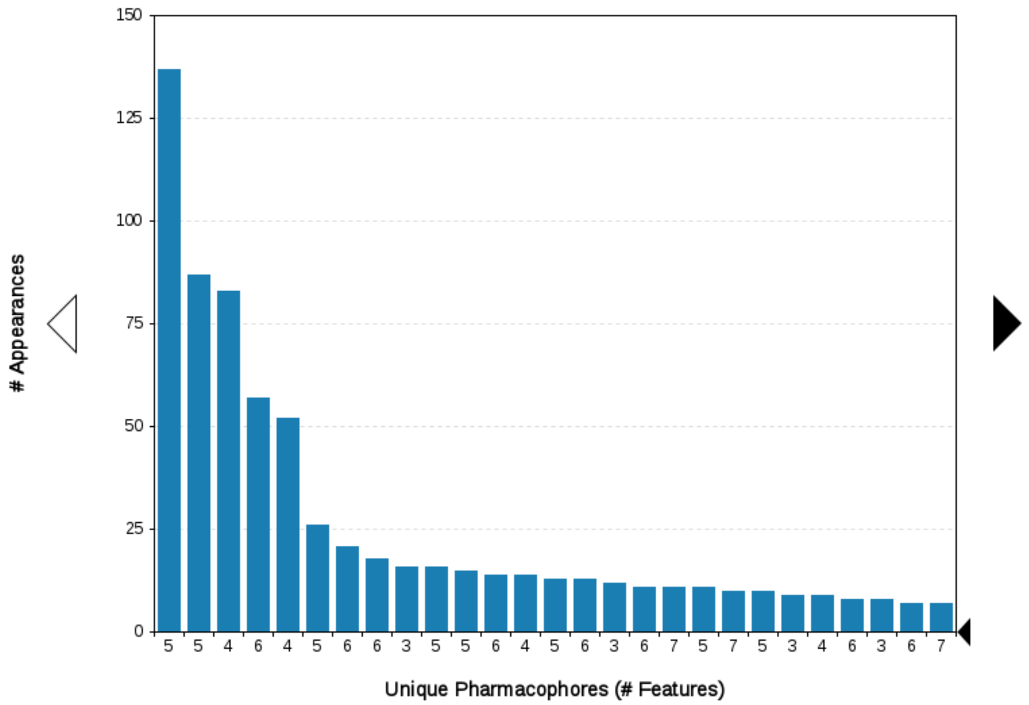

Figure S4. Plot of the most frequent unique structure-based pharmacophore models derived from the molecular dynamics simulations of the OGT in complex with Vs-51. The numbers below the bars indicate the numbers of interaction features observed during molecular dynamics simulation for the pharmacophore models.

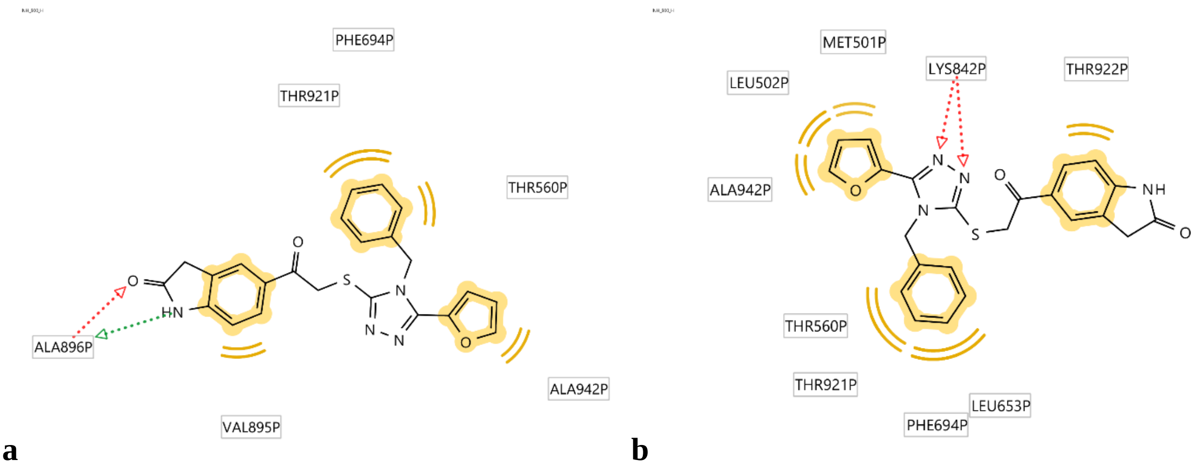

Figure S5. Schematic representation of the interactions between OGT and Vs-51 observed in **a**) the first and **b)** the second most frequently occurring structure-based pharmacophore model.

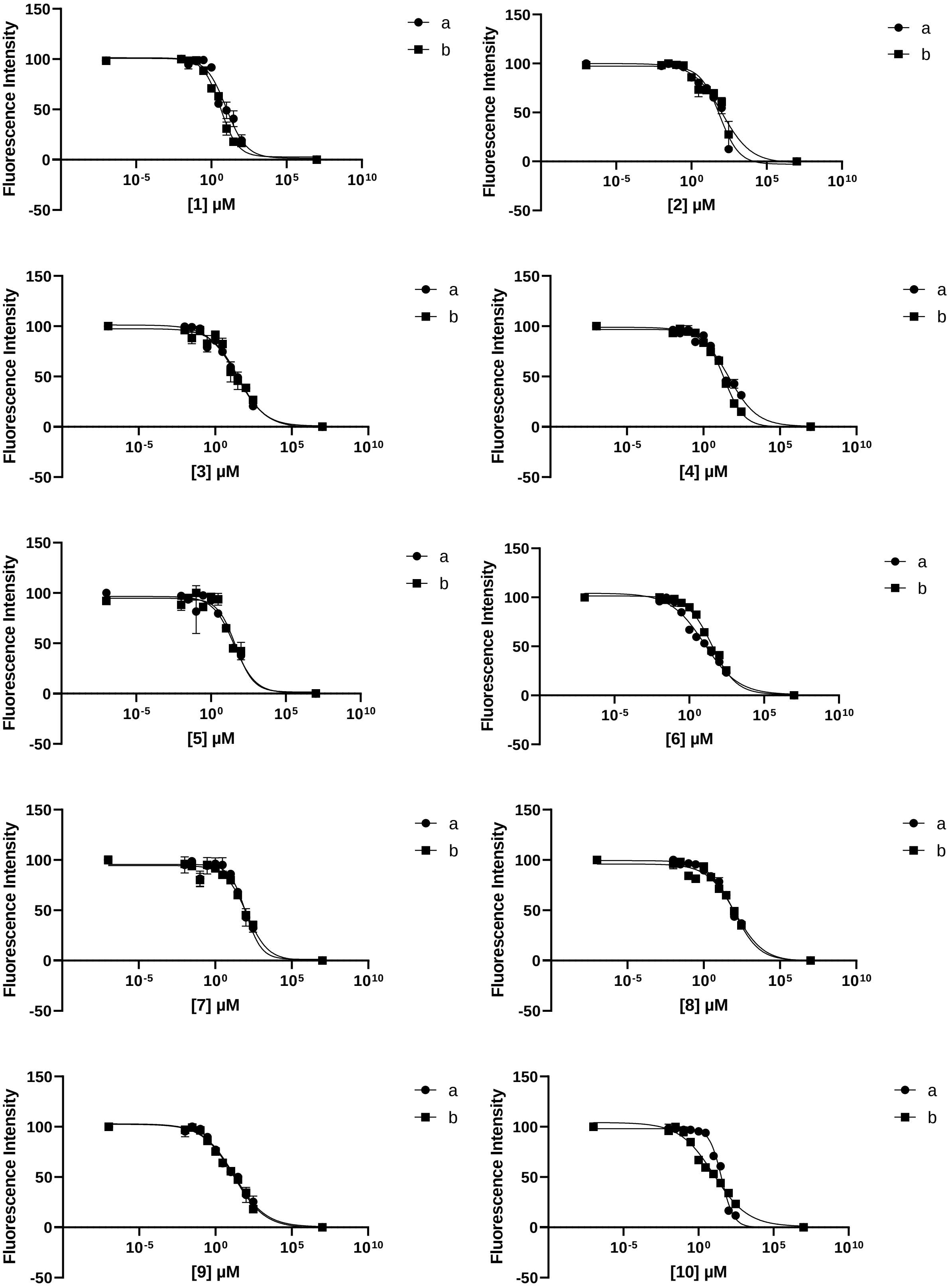
Fluorescence Intensity
150
100
50
0
-50
10^{-5}
10^{0}
10^{5}
10^{10}
a
b
[1] μM
[2] μM
[3] μM
[4] μM
[5] μM
[6] μM
[7] μM
[8] μM
[9] μM
[10] μM

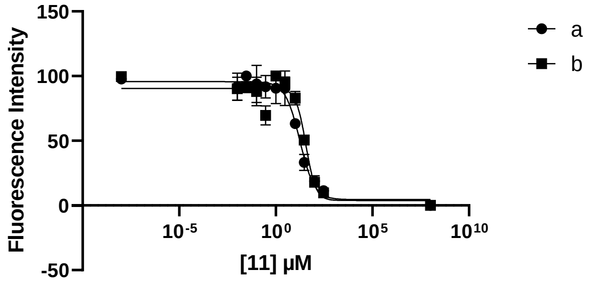

Figure S6. IC_{50} curves of compounds **1-11** measured in two independent experiments. Normalized fluorescence intensity values are expressed as a percentage of the DMSO control.

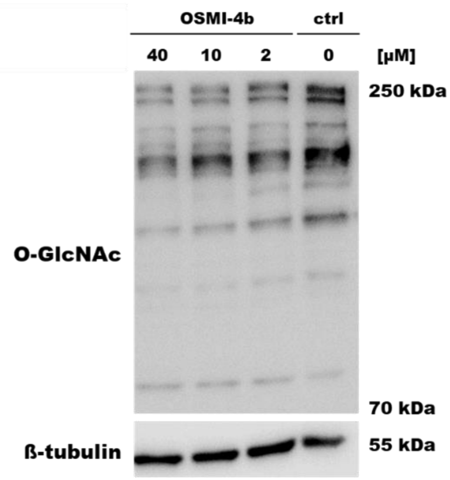

Figure S7. Representative picture of western blot analysis of O-GlcNAc levels after treating AMO1 cells with OSMI-4b for 4 hours (two independent experiments).

Compound **1** ^{1}H NMR

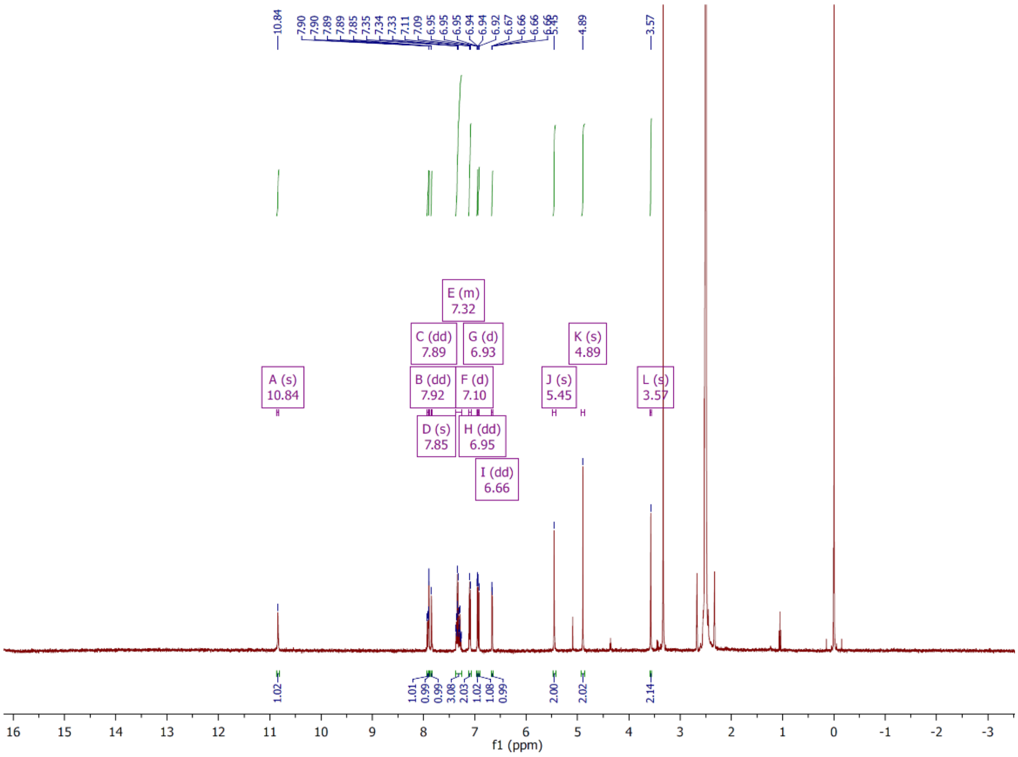

Compound **1** ^{13}C NMR

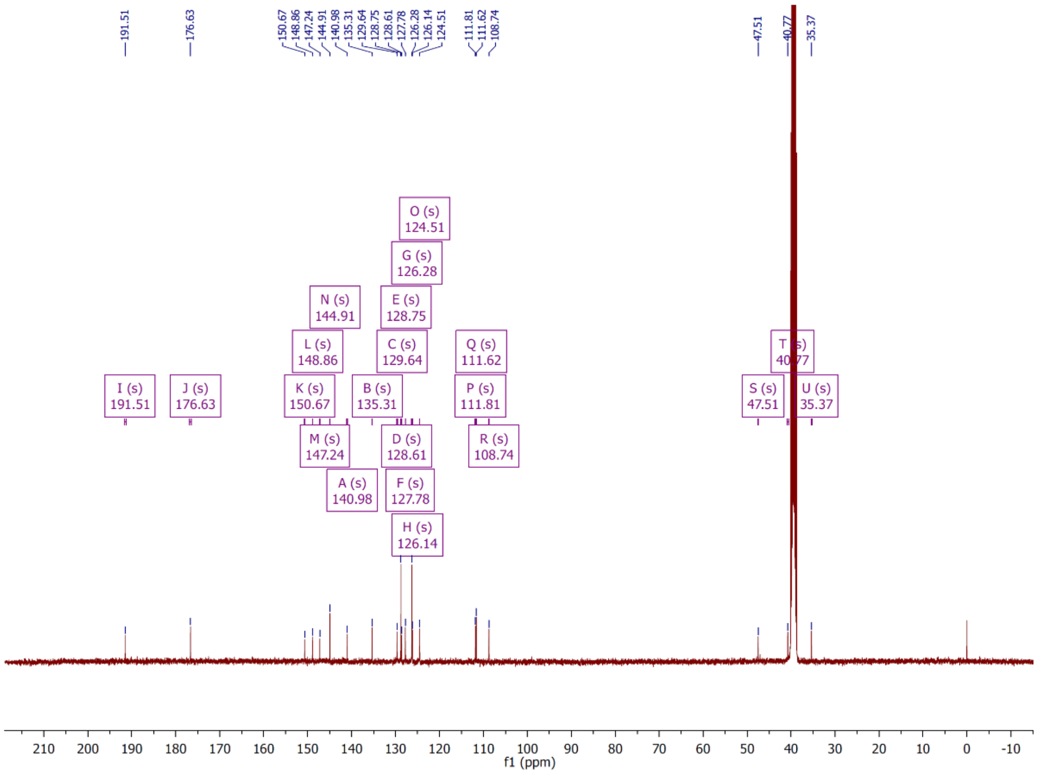

Compound **2** ^{1}H NMR

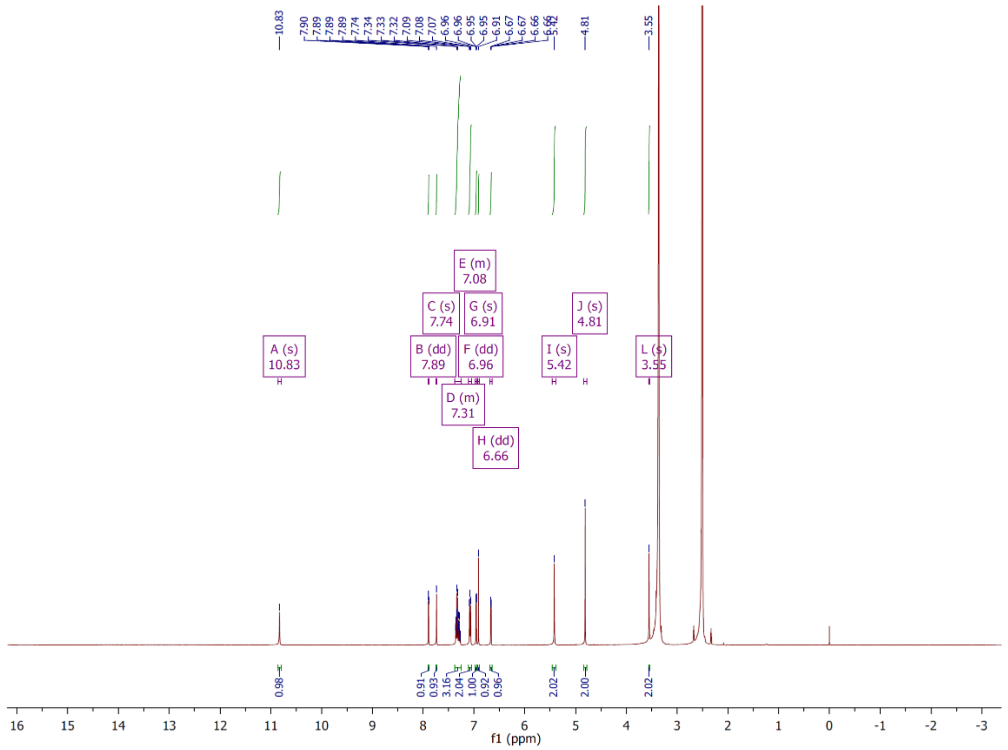

Compound **2** ^{13}C NMR

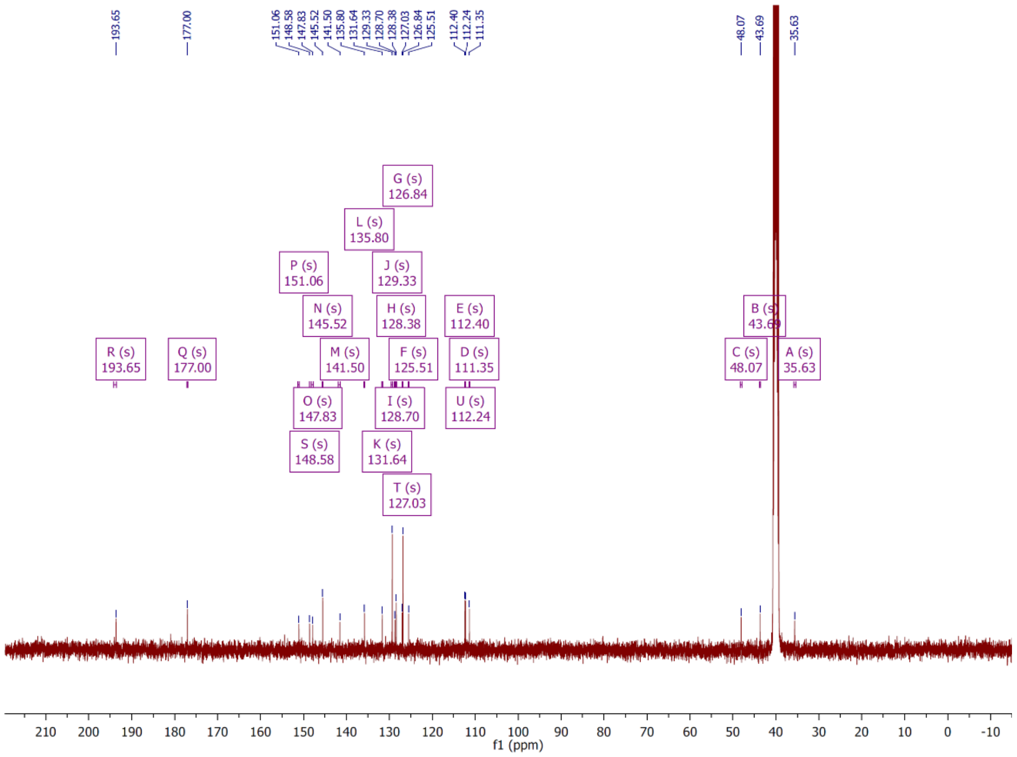

Compound **3** ^{1}H NMR

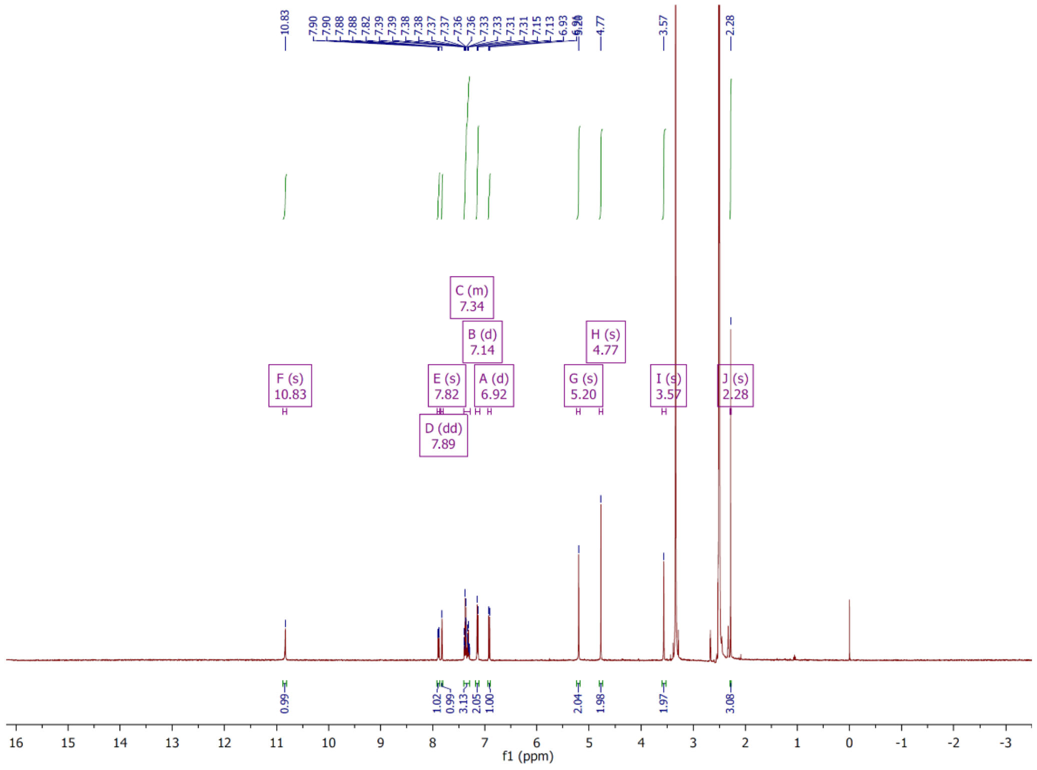

Compound **3** ^{13}C NMR

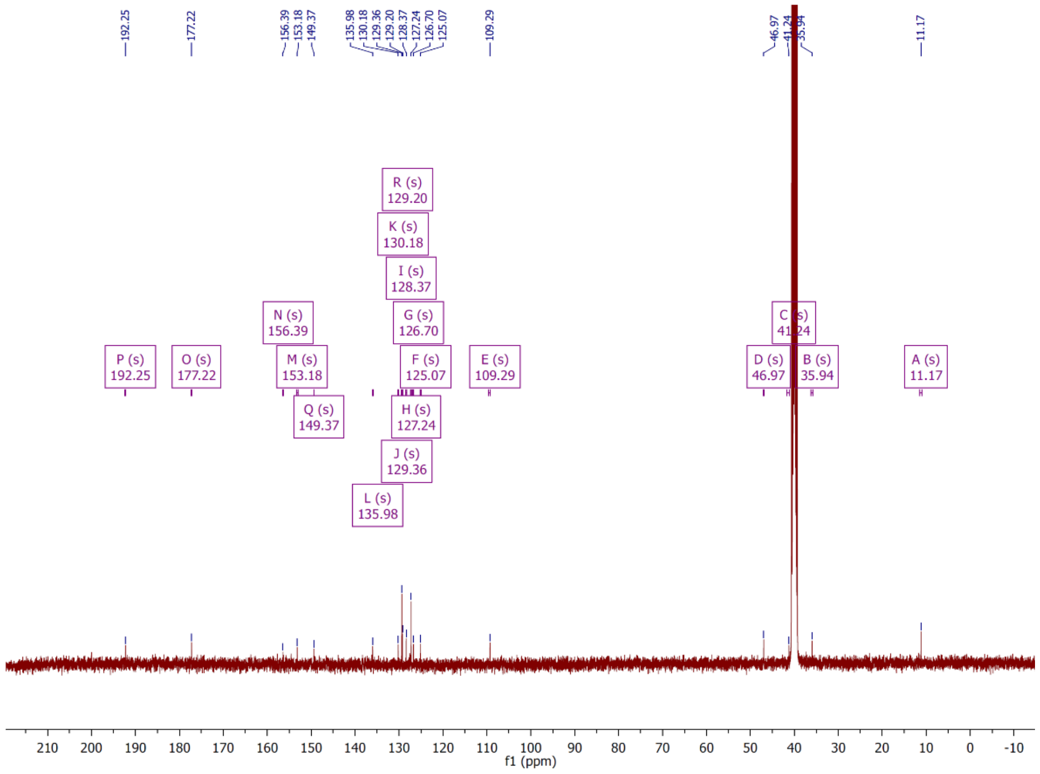

Compound **4** ^{1}H NMR

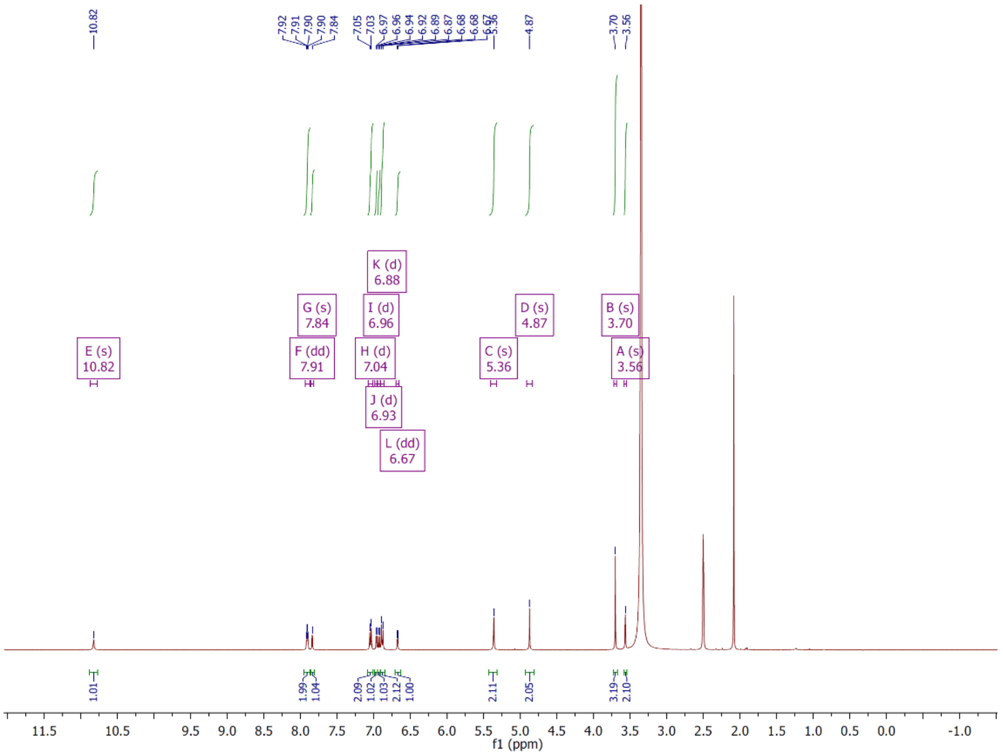

Compound **4** ^{13}C NMR

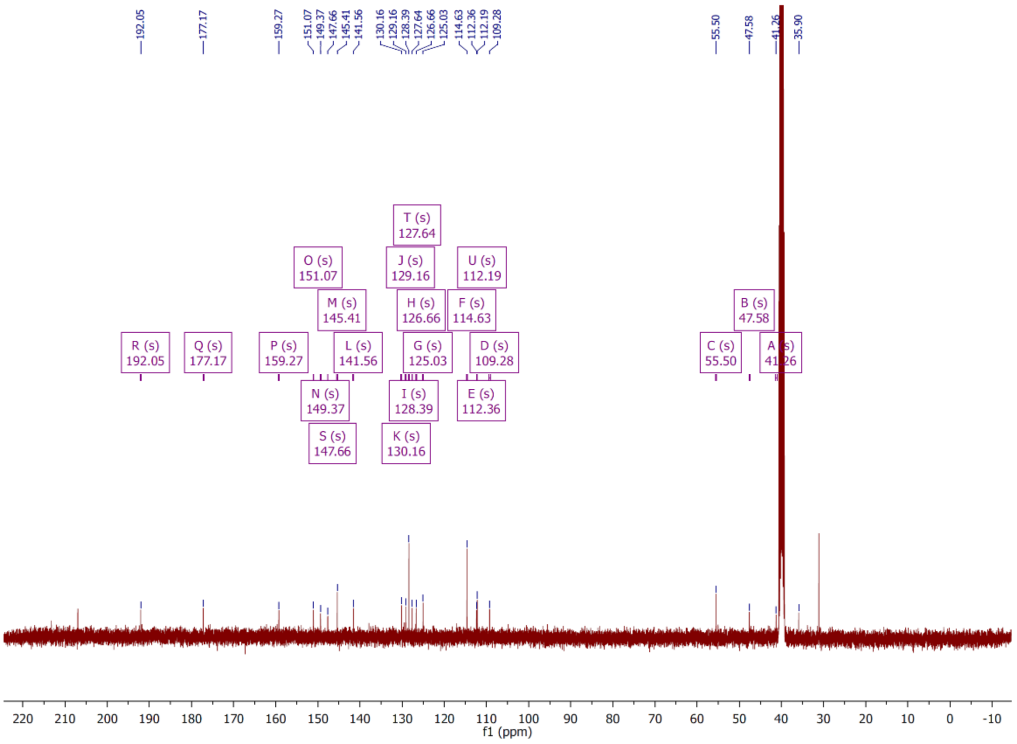

Compound **5** ^{1}H NMR

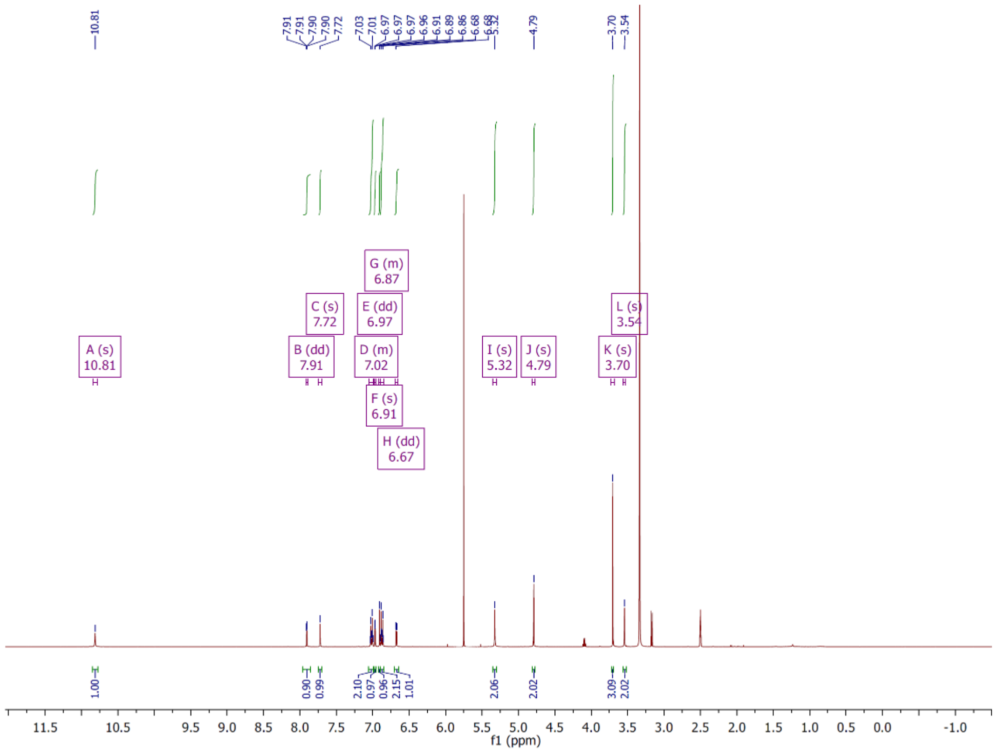

Compound **5** ^{13}C NMR

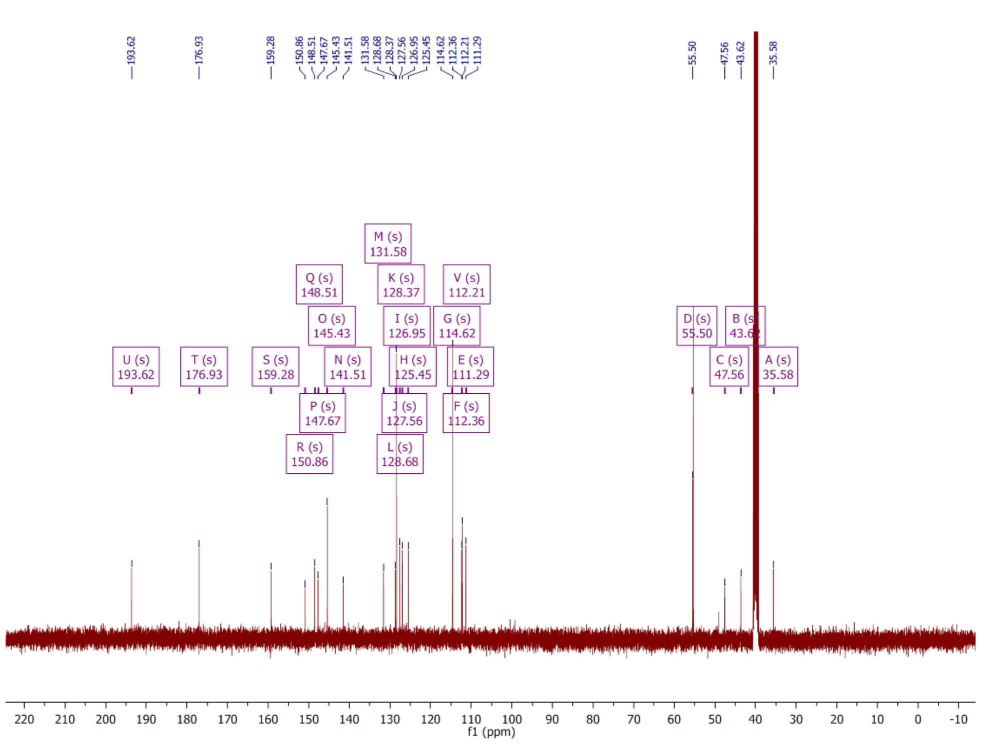

Compound **6** ^{1}H NMR

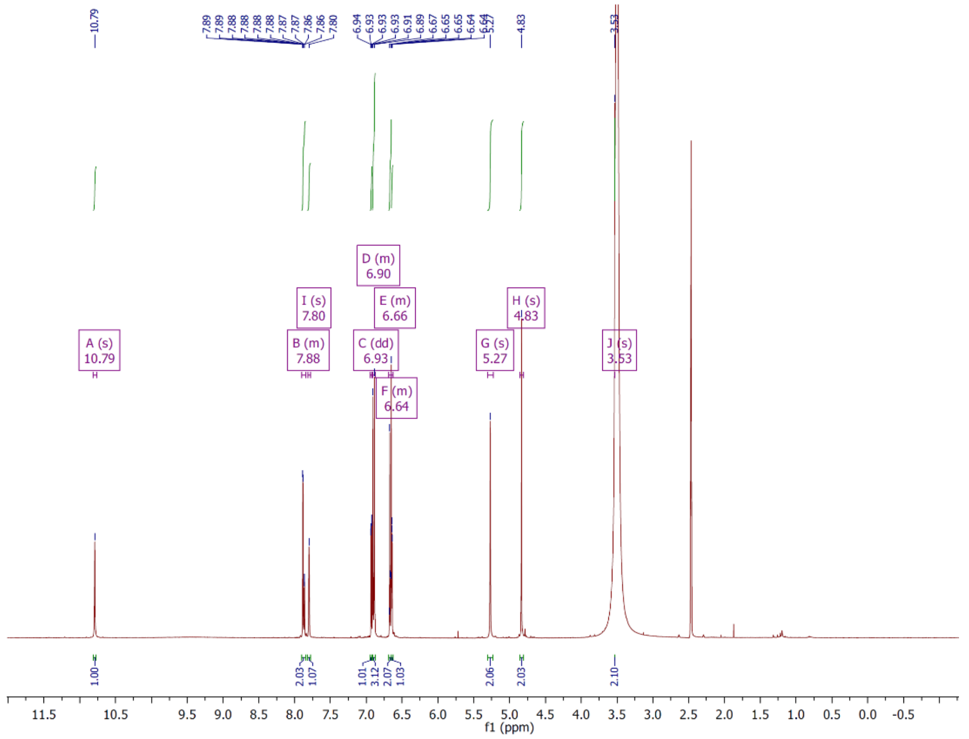

Compound **6** ^{13}C NMR

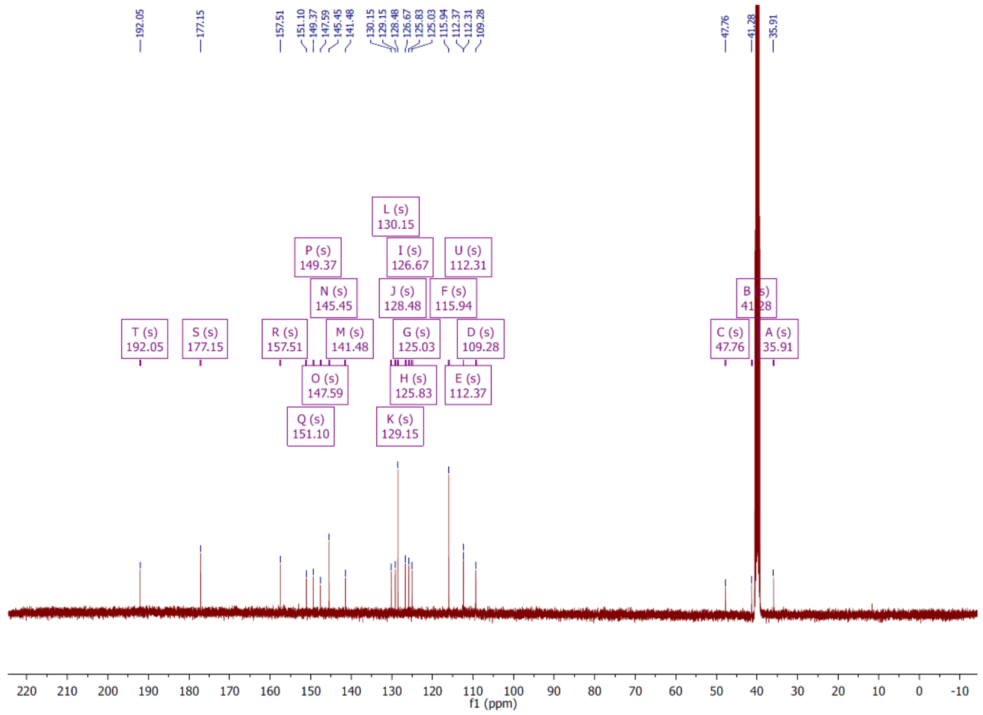

Compound **7** ^{1}H NMR

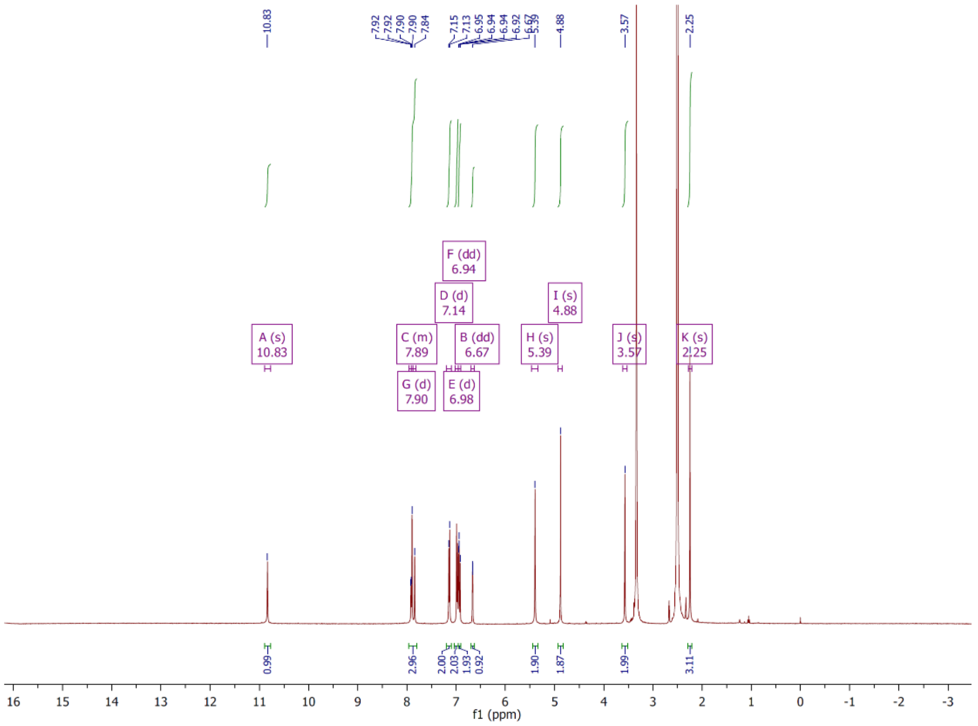

Compound **7** ^{13}C NMR

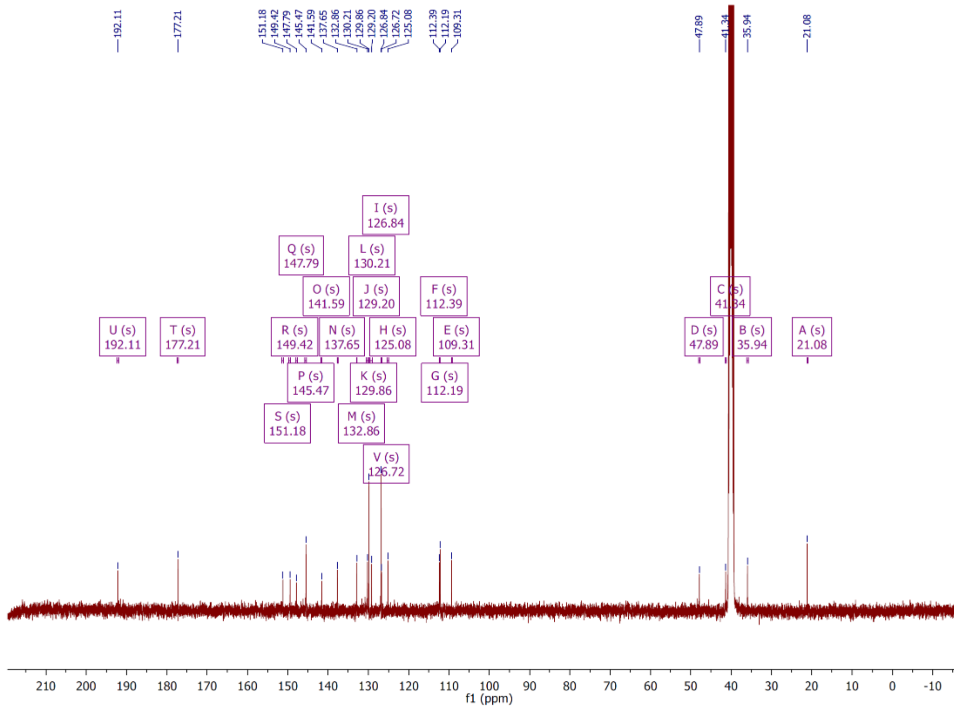

Compound **8** ^{1}H NMR

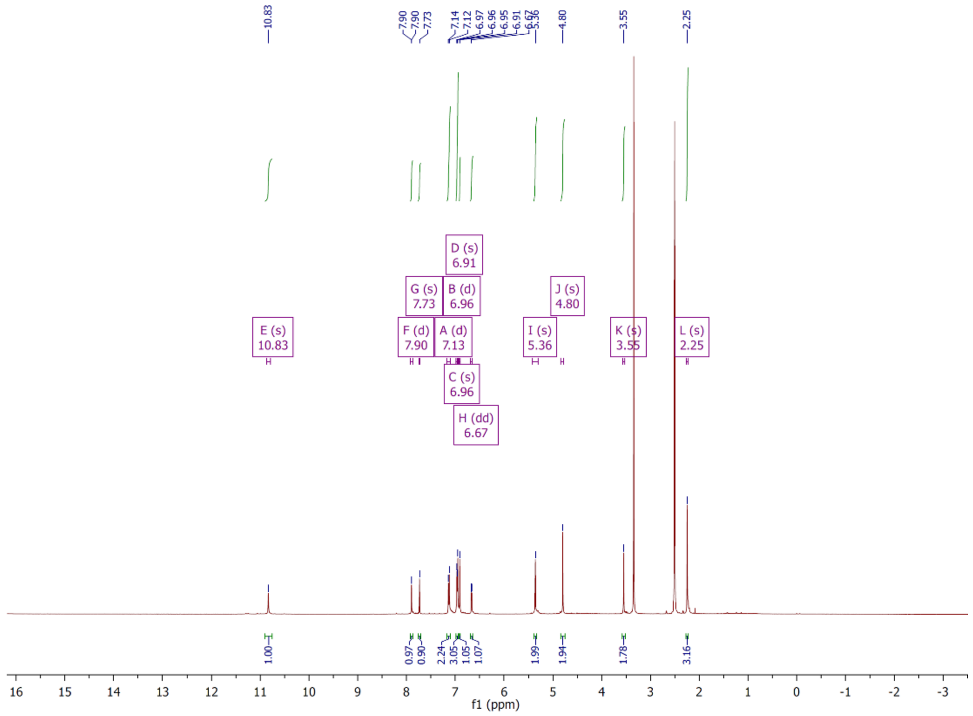

Compound **8** ^{13}C NMR

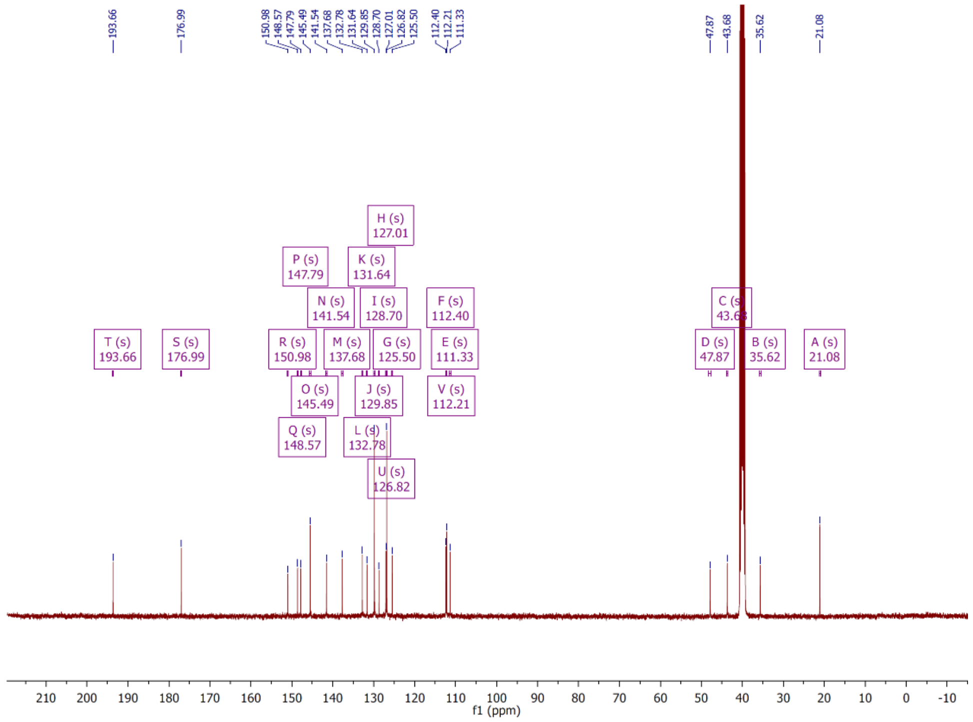

Compound **9** ^{1}H NMR

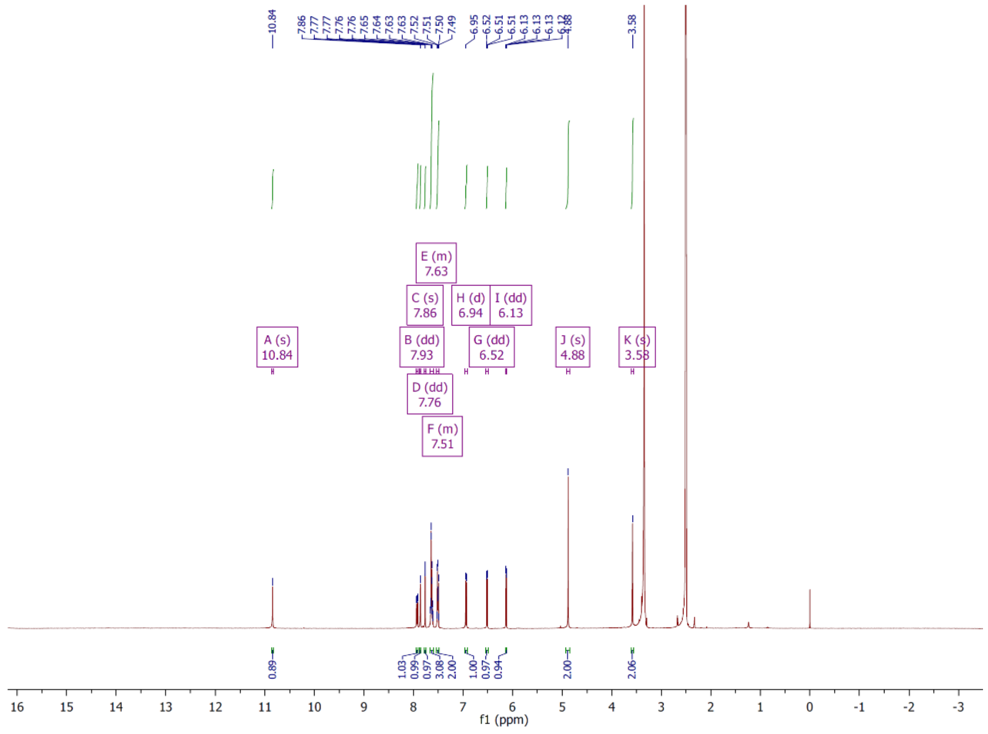

Compound **9** ^{13}C NMR

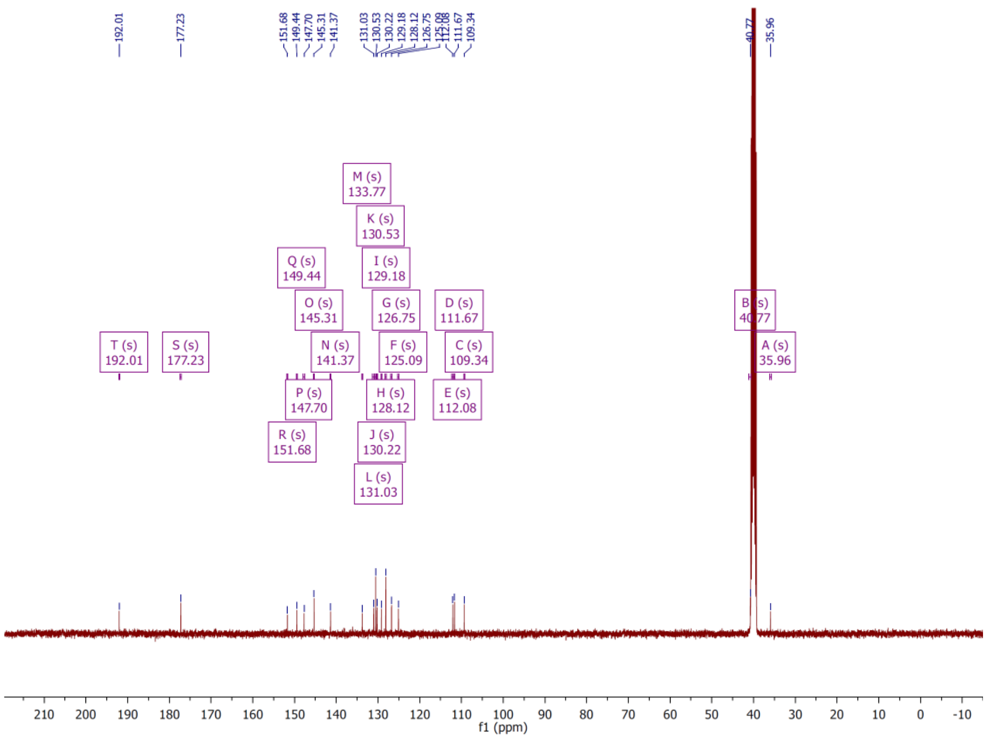

Compound **10** ^{1}H NMR

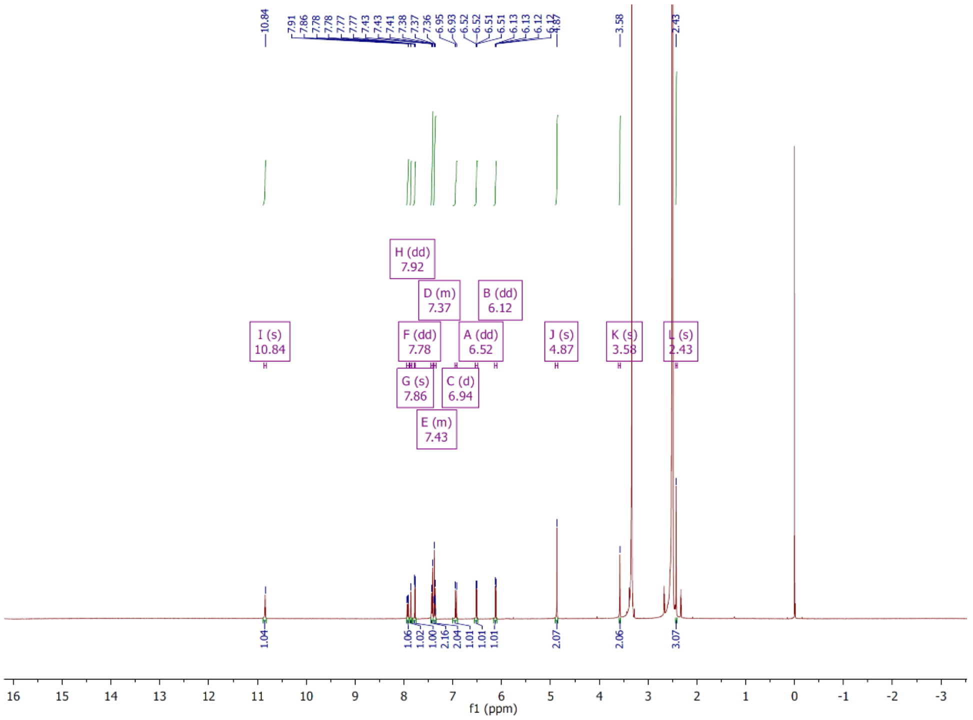

Compound **10** ^{13}C NMR

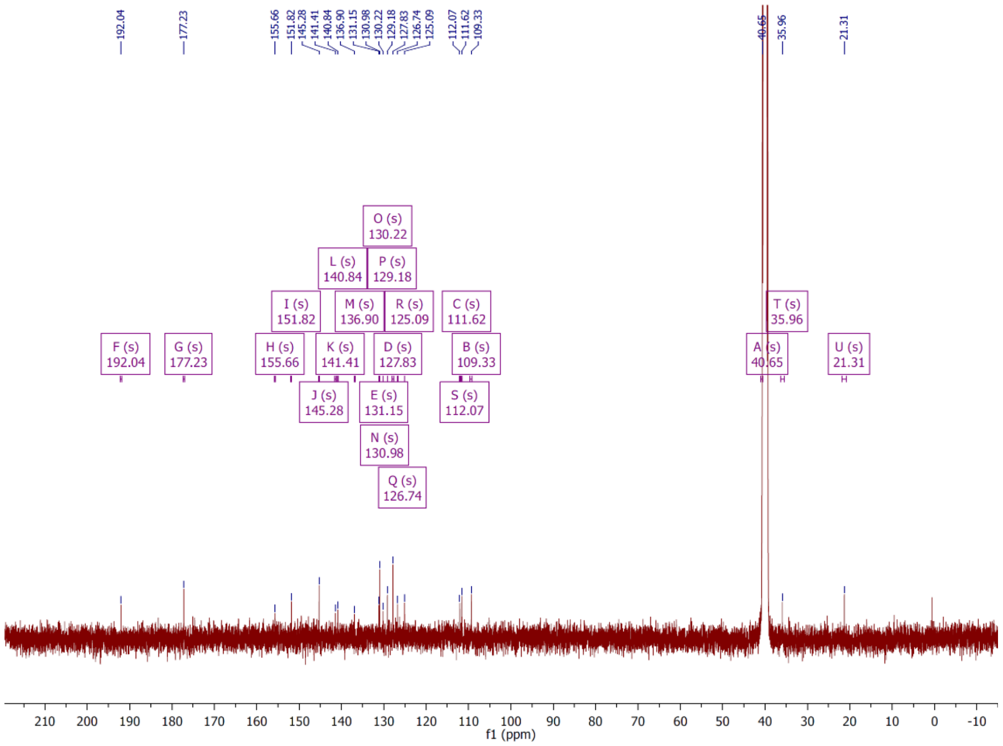

Compound **11** ^{1}H NMR

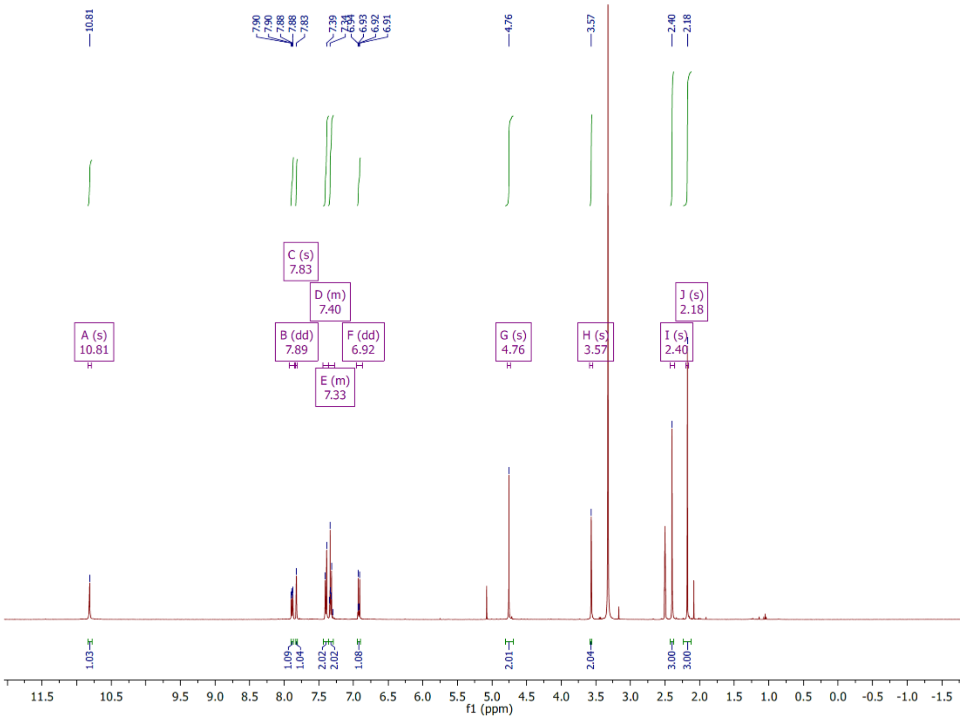

Compound **11** ^{13}C NMR

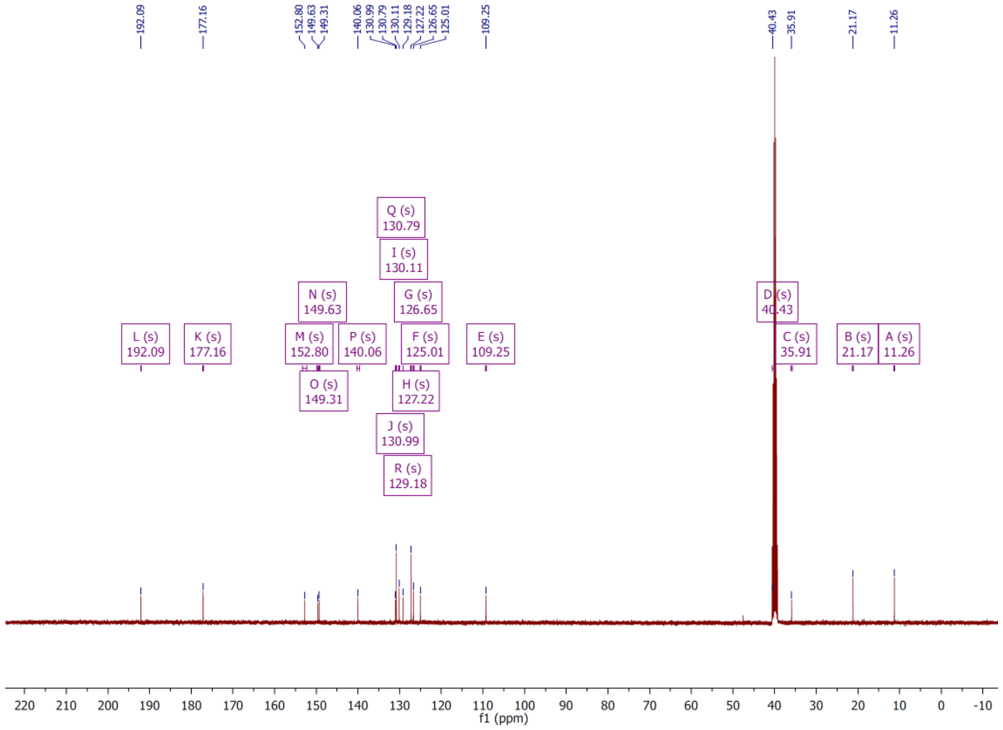

Compound **12** ^{1}H NMR

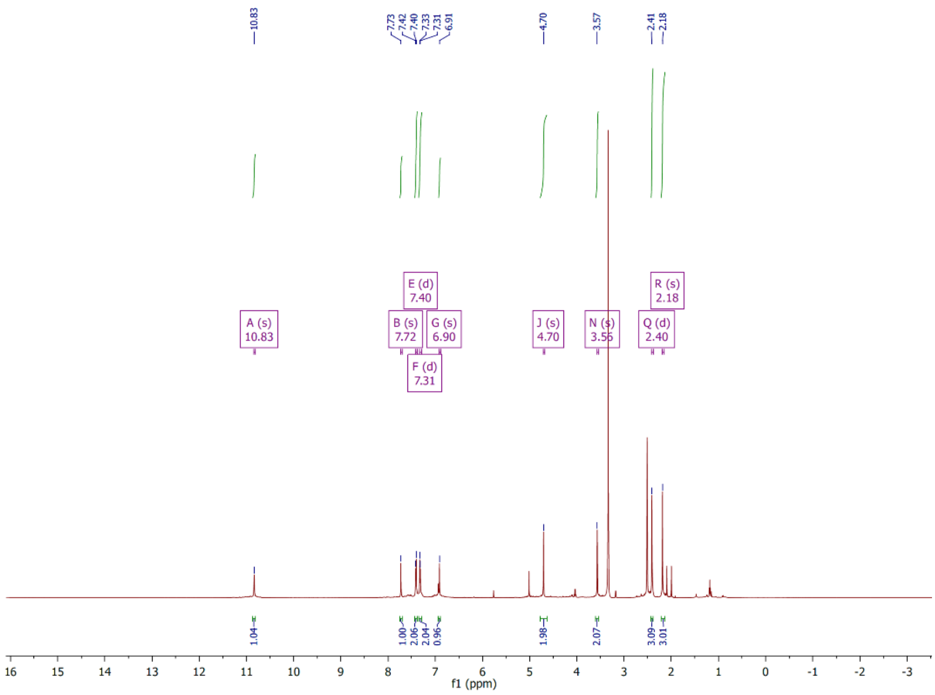

Compound **12** ^{13}C NMR

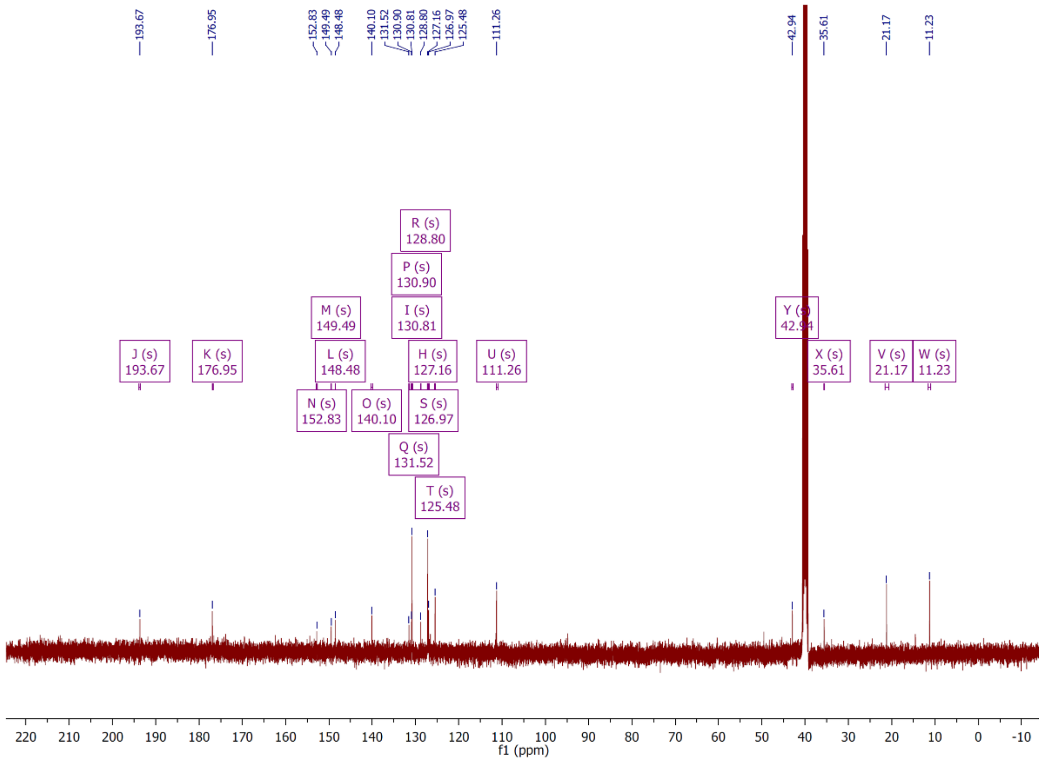

Compound **13** ^{1}H NMR

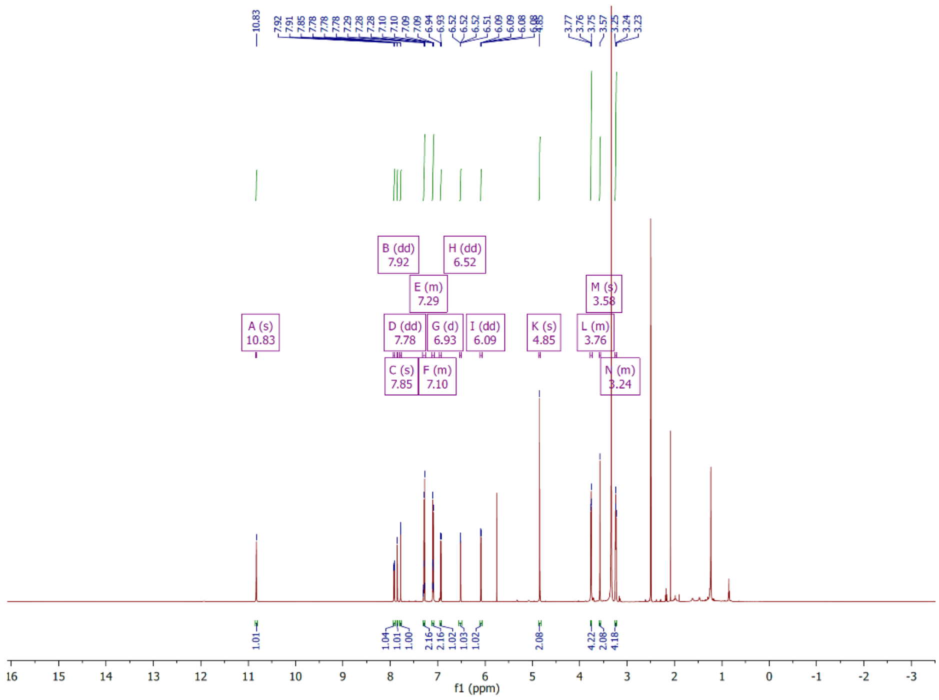

Compound **13** ^{13}C NMR

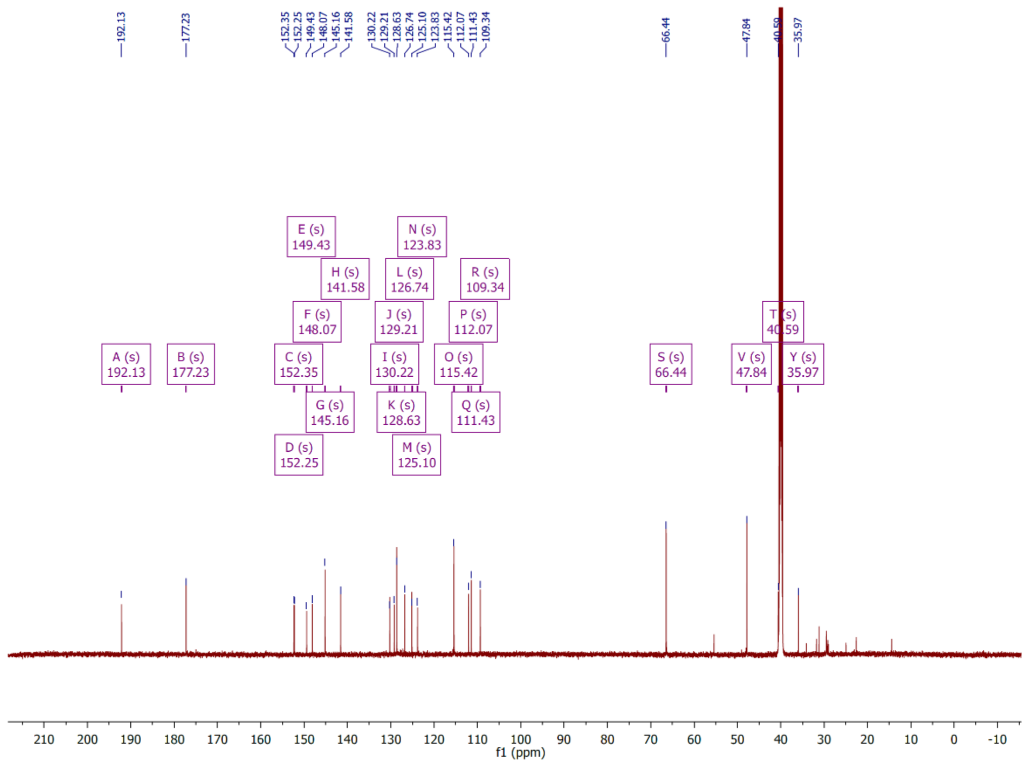

Supplement: Supplementary file 1 [file molecules-27-01996-s001.zip › molecules-1612960-supplementary.pdf]
